# Forensic psychiatry in Finland: an overview of past, present and future

**DOI:** 10.1186/s13033-020-00362-x

**Published:** 2020-04-16

**Authors:** Allan Seppänen, Petteri Joelsson, Aulikki Ahlgren-Rimpiläinen, Eila Repo-Tiihonen

**Affiliations:** 1 Psychoses and Forensic Psychiatry, Helsinki University Hospital, Helsinki, Finland; 2 Vanha Vaasa Hospital, Vaasa, Finland; 3 National Institute for Health and Welfare, Forensic Psychiatry, Helsinki, Finland; 4 University of Eastern Finland, Kuopio, Finland

**Keywords:** Forensic psychiatry, Finland, Forensic assessments

## Abstract

Despite a recent contrary trend, Finland has been for decades one of the most violent countries in Western Europe. Also, Finland has had one of the highest number of psychiatric beds per capita in Europe, although this, too, has seen a sharp decline. Against this background, among other national idiosyncrasies, Finland has developed its forensic psychiatric services. Here, we describe the legal, organizational and clinical structure of these services, and outline the historical and current issues that have shaped them. Finally, we consider future challenges facing the Finnish forensic service system, as part of wider European and global trends.

## Introduction: violence in Finland

Finland (ind. 1917) is a Northern-European urbanized parliamentary democracy and a member of both the OECD and the EU, with a total population of approximately 5.4 million, and which usually scores high in indicators such as peacefulness, stability and quality of life [1]. Similarly to other Nordic countries, Finnish health services are publicly funded and the general access to these services is considered good. On these grounds, it may come as a surprise that Finland is, in fact, one of the most violent countries in Western Europe [2]. Indeed, according to an unbroken series of statistics from the mid-1750s onwards [3], in Finland homicide rates have been considerably higher and more volatile than in the other Western European and Nordic Countries. In Finland, the period of industrialization was accompanied by a more or less permanent increase in violent crime, whilst elsewhere in Western Europe homicide rates decreased [4]. Yet, in recent years, homicides have become less frequent in Finland too: in 2017 the lowest rate/capita ever—1.11/100 000—was recorded [3]. In comparison, in 1918, a year after Finland gained independence, the recorded homicide rate was > 60/100,000 as the country was recovering from civil war. The rate remained similar well into the 1930s, after which it began to decline, only to peak again after World War II [2].

In addition to violence-inducing historical and cultural factors, such as the widespread civil unrest and prohibition laws of the early twentieth century, the latter arguably contributing to a culture of binge- drinking still prevalent today [5, 6], demographic features specific to Finland need to be taken into account. Immigration to Finland has remained low compared to other West European countries. Also, migration within the country has historically been low and distinct genetic differences have developed between the populations living in the western and eastern parts of the country [7]. Finland is sparsely populated and the population lives more rurally than that in Western Europe despite gradual urbanization. Accordingly, unique genetic features of the Finnish founder population have contributed to violent crime in Finland. For instance, there are genotypes enriched to the Finnish population which predispose to severe impulsivity (a stop codon in HTR2B) [8] or committing severe recidivistic impulsive violent crimes when exposed to heavy drinking and childhood physical abuse (MAO-H allele) [9]. Indeed, the majority of Finnish homicides occur in the context of drinking quarrels between unemployed, middle-aged male alcoholics, i.e. a so-called “ryyppyriitatappo” (homicide during a drunken quarrel). For instance, during the period 2010–2015, in 67% of all homicides all persons involved were intoxicated and in 82% of the crimes at least one of the persons involved was intoxicated [4].

## Historical developments

### General psychiatric services

Between the years 1809 and 1917 Finland was a Grand Duchy of Russia with its own parliament and civil administration. According to the law passed in 1840, psychiatric hospitals were separated from other hospitals and thus the first specific psychiatric hospital—Lapinlahti hospital—was founded in Helsinki in 1841. Since the beginning of the twentieth century and particularly after independence, local administration began increasing the number of hospital beds. The amount of hospital beds peaked in the 1970s, reaching over 4 beds for 1000 inhabitants, which was one of the highest in Europe [10]. Deinstitutionalization began in the 1980s as the focus of treatment transferred from hospitals to outpatient care. The development of antipsychotic medications and the need for treating also non-psychotic disorders contributed significantly to this trend. In the 1990s psychiatric organizations started yet again to integrate with general hospitals [11]. The current general psychiatric bed provision is ca. 0.6/1000 [12].

### Forensic psychiatric examinations

The first forensic examinations in Finland were conducted by prison doctors in the 1830s and the first hospital to conduct forensic examinations was Lapinlahti hospital in 1841. However, despite this emerging practice of forensic examinations, the concept of decreased criminal responsibility due to mental illness was properly embedded into law only after 1889 [13]. Yet in the beginning of the twentieth century still only a few examinations were conducted annually. After independence, the amount of annual examinations began increasing together with the number of hospital beds nationwide. Also, the first vacancy for a forensic psychiatrist was founded in Lapinlahti 1918. After WW2, the number of annual examinations rose to approximately 200, peaking at ca. 300 in the late 80s [14, 15].

### Forensic hospitals

Niuvanniemi Hospital has been operating since 1885 and Vanha Vaasa Hospital since 1768, the latter as a general hospital until 1931. Their role as forensic psychiatric hospitals developed by degrees; the number of forensic patients in these hospitals was increased dramatically in the 1930s, and their current status as specialist state forensic hospitals operating under the Ministry of Social Affairs and Health was cemented by the reforms in mental health legislation of 1952 [16–18]. The current unit of Enhanced Rehabilitation and Forensic Psychiatry within Helsinki University Hospital, on the other hand, was established as late as 2015 by incorporating and reformatting units located at the site of the century-old Kellokoski Hospital north of Helsinki. A psychiatric hospital specifically for prisoners has also been operating within the prison system since 1911 in Turku [14] and later also in Vantaa.

### Academic contributions

The first book published in Finland dealing with forensic psychiatric issues was by Dr. Theodor Löfström (1857–1907) in 1901 [19]. Later, in 1910s and 1920s, Dr. Akseli Nikula (1884–1956) continued to diversify the forensic literature in Finland by publishing on topics such as fratricide, mass criminality and psychiatric presentations associated with criminal behavior [20]. Professor Martti Kaila (1900–1978) continued to develop Finnish forensic psychiatry with his seminal work on adolescent offenders [21, 22]. Furthermore, Dr. Panu Hakola (1932-), who was later to become the first professor of forensic psychiatry in Finland (see below), completed his thesis on polycystic lipomembranotic osteodysplasia with sclerosing leukoencephalopathy, also known as Nasu-Hakola disease, in 1972 [23]. As several cases of Nasu-Hakola disease had committed violent and sexual offences due to the disease affecting the frontal lobes, the thesis contributed to our understanding of the neurophysiological basis of behavior regulation.

More recently, there has been an increasingly wide array of contributions by Finnish forensic psychiatrists to the scientific literature, ranging from biological psychiatry [24–29] and pharmacological interventions [30–32] to the psychiatric epidemiology of violence [33, 34] and forensic nursing and rehabilitation [35–39], to mention but a few.

The first chair of forensic psychiatry in Finland was established in 1983 at Kuopio University [16] (later reorganized and -named as the University of Eastern Finland), in affiliation with Niuvanniemi Hospital. Panu Hakola, MD, PhD, was appointed as the first professor and chairman, and he continued for 12 years in that position, whilst continuing as medical director for Niuvanniemi Hospital. He continued to consolidate the strive for clinical, academic and organizational excellence in Finnish forensic psychiatry [16], which continues today. Now, forensic psychiatry is in Finland an established, independent medical specialty with a six-year training program. Ca. 60 doctors nationally hold the specialist degree.

## Current legislation

Laws of particular relevance for psychiatry in Finland are the Mental Health Act (1990), and, insofar as it pertains to forensic psychiatry, the Criminal Law (1889), the Law on State Mental Hospitals (1987 and 1997) [40] and Law on the Care of the Mentally Retarded (1977).

In terms of forensic psychiatric assessments, Finnish law (Code of Judicial Procedure 17 section, 37 paragraph) stipulates that the criminal court can order a forensic psychiatric examination to take place if

1. The accused is shown to have committed a crime that is punishable as a criminal offence (11 Sect. 5a §) and.
2. A forensic mental evaluation can be justified, and.
3. The accused is willing to be examined, or he is held prisoner, or he is accused of a crime that is punishable by a prison sentence of more than one year (homicide, a felonious assault etc.).

Under certain preconditions, the examination can also be performed by order of the court of appeals, or while the pretrial investigations are still ongoing. In any case, within the Finnish legislative context, the primary issue that the forensic psychiatric assessment must consider is the question of criminal responsibility. According to Finnish criminal law (39/1889), the perpetrator of a crime is not criminally responsible if, at the time of the crime, he was not able to understand the factual nature or unlawfulness of his act, or his ability to control his behavior was decisively weakened due to mental illness, severe mental deficiency, a serious mental disturbance, or a serious disturbance of cognition. Also, if a person is not irresponsible according to this definition, but his ability to understand the nature of the act or its illegality or his ability to control his actions was, due to the same reasons, severely diminished, this can be taken into account when passing sentence and can result in a less severe sentence due to diminished responsibility [41].

**Fig. 1:**
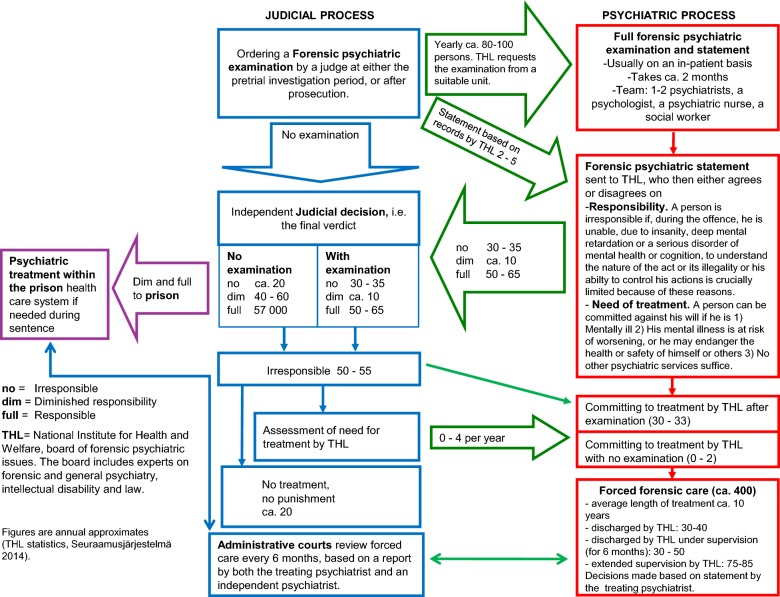
Forensic psychiatric system in Finland

Thus, during court procedures, the judge may decide, according to the criteria listed above, that a forensic psychiatric report is needed before judgment can be passed. In less serious cases, particularly if the accused is already undergoing psychiatric treatment, a report from the treating psychiatrist will suffice. In more serious cases, namely violent and sexual crimes, THL (National Institute of Health and Welfare) is usually requested to arrange a full forensic examination (see Fig. 1). The examination report must stipulate.

1. Relevant diagnoses, primarily psychiatric and neurological.
2. The level of responsibility in respect to the crime(s) of which the person is accused.
3. Whether the criteria for compulsory psychiatric treatment, as defined by the Finnish Mental Health Act, are fulfilled.
4. Fitness to be heard at trial.

## Forensic examinations today

**Fig. 2:**
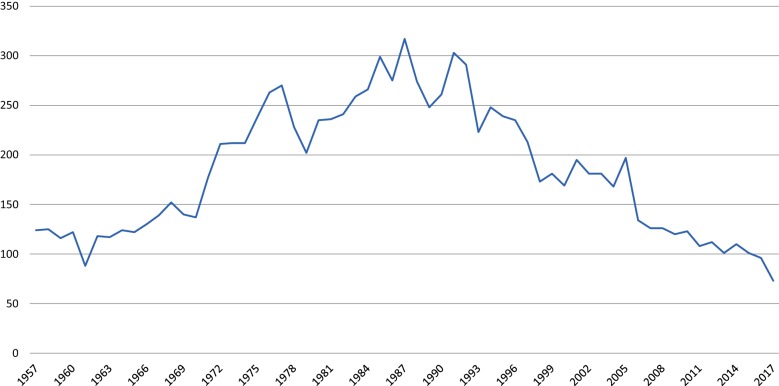
The number of forensic psychiatric examinations since 1957

Forensic psychiatric examinations usually take place in a psychiatric ward environment at the two state forensic hospitals, Niuvanniemi Hospital and Vanha Vaasa Hospital, the forensic psychiatry units of university hospitals, or the prison mental hospital. Full forensic psychiatric examinations are currently produced at the annual rate of ca. 80–100, having decreased from ca. 300 during the previous decades (Fig. 2) [42]. The process takes a maximum of 2 months and is conducted by a specialist in either forensic psychiatry or general adult psychiatry. The multidisciplinary examination team also includes a psychologist, social worker and a psychiatric nurse, and other experts can be consulted accordingly. The examinee is subject to structured and unstructured interviews, psychological tests, constant surveillance and various radiological scans and lab-tests [43].

With the full forensic psychiatric examination completed, a report, which must include the examinee’s own opinion of the report and its conclusions, is sent to The National Board for Forensic Psychiatric Affairs (NBFPA) at THL. The NBFPA is nominated by the Ministry of Social and Health care for 4 years at a time and consists of a chairman, two experts on psychiatry and an expert on law. An expert qualified in matters concerning intellectual disability can be called to attend the session if needed. The NBFPA then gives its own statement, based on the examination report and relevant legal documents, to the court. The court decides independently on the final verdict, including the level of responsibility, although usually the court is in agreement with the forensic psychiatric experts. If the person fulfills the criteria for involuntary psychiatric care [41, 44] when the forensic mental examination is completed, the NBFPA issues the treatment order for forensic psychiatric care, irrespective of the level of responsibility at the time of the offence.

Annually 1-5 persons with intellectual disabilities are involved in the forensic evaluation process. Also in these cases THL issues the order for involuntary care when considered necessary, but regional authorities with expertise on developmental disorders determine the appropriate specialized care program and the institution where the care will take place.

**Fig. 3:**
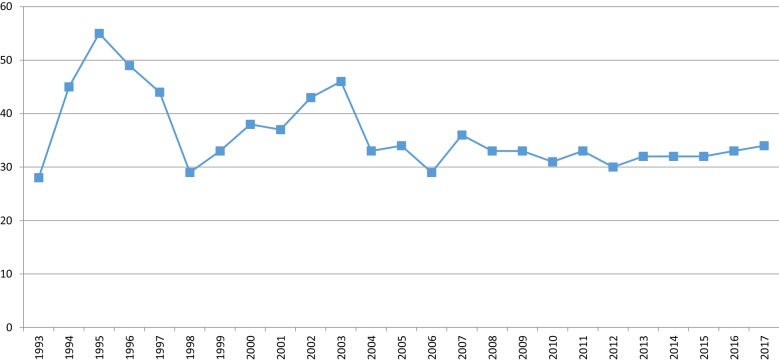
Treatment orders for irresponsible offenders since 1993

**Fig. 4:**
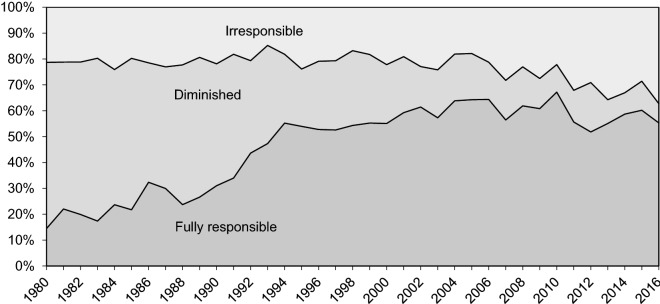
Criminal responsibility since 1980

About 30–35 offenders are annually committed to involuntary forensic treatment. Interestingly, this number has not changed despite fewer examinations being conducted (Fig. 3) [42]. Instead, the number of examinees deemed to have diminished responsibility has decreased dramatically (Fig. 4). This is mainly due to a change in how personality disorders have been viewed in terms of criminal responsibility; before 1990s a diagnosis of personality disorder, particularly borderline and antisocial, was commonly used to justify diminished responsibility. However, this was seen as problematic and had, in fact, been criticized for decades [45, 46], since it resulted in the offenders with the highest risk of re-offending routinely getting diminished prison sentences [13]. What is more, it was found that those assessed fully or partially responsible did not, in fact, differ diagnostically from each other as groups [14, 47]. Also, since a legal reform in 2004, diminished responsibility has not in itself sufficed to justify a decreased sentence [48], arguably rendering the prospect of a forensic examination less attractive to offenders.

In addition to the forensic examination described above, the court must request an assessment of dangerousness when considering sentencing a serious recidivist to a so-called combination sentence, i.e. a sentence that includes a year-long supervised probation period after a prison sentence. The assessment of dangerousness must include a structured professional risk assessment, such as PCL-R and HCR-20. The law requires the assessment report to state whether the examinee should be considered dangerous to the life, health or freedom of others [43].

## Forensic treatment

The treatment of forensic psychiatric patients is most often commenced in the forensic state mental hospitals, which have combined ca. 450 beds. The length of treatment varies, but is almost invariably several years, 10 on average, including the pre-discharge outpatient period. The total length of treatment is not limited by law but, instead, by clinical progress. Both the committing and discharge process is administered by healthcare authorities at THL, under the Ministry of Social Welfare and Health, based on reports from the treating unit. As in all cases of involuntary treatment, the provincial administrative courts review and reinforce the commitment at regular intervals, which, in the case of forensic patients, is 6 months and which also involves an expert psychiatric statement independent from the treatment facility.

Prior to final discharge, a forensic patient is typically released from hospital for provisional outpatient treatment, on conditions determined by THL, for a maximum of 6 months at a time. During this period the patient is under the supervision of a psychiatric unit of the local hospital district, and, whilst continuing his treatment as an outpatient, he legally still remains in involuntary psychiatric care. He is required to meet a psychiatrist every month, who in turn reports to the psychiatric hospital that is responsible for the involuntary hospital care. The patient can be readmitted to hospital at any given time, if the need were to arise [49].

At the point where the patient no longer fulfills the criteria for involuntary psychiatric care (e.g. the patient’s psychiatric condition is deemed to be stabilized and the risk for reoffending is assessed as being sufficiently low), the hospital is obliged to terminate the involuntary treatment and subordinate this decision to NBFPA. NBFPA bases the final decision on the termination of treatment on, for instance, how well the patient has adhered to his treatment plan and risk management strategy during the provisional outpatient period. After the provisional outpatient period successfully ends, the patient’s legal status as a forensic patient is terminated and he has no special obligations to adhere to.

## Current trends and reflections on the future

Finland is currently preparing for a major social welfare and healthcare reform. This involves a major overhaul of service structures, including the creation of autonomous bodies for the purpose of organizing social welfare and healthcare services in their respective geographical areas. However, certain services will still be organized in a highly centralized way, including forensic psychiatry. Therefore, it seems that there is relatively little pressure in Finland to move toward smaller forensic units located within their own catchment areas, such as has been the case in the UK and Italy [50, 51]: arguably upholding a centralized specialist forensic service is justified in Finland by a much smaller population.

Although the welfare and healthcare reform will thus leave the forensic services relatively unaffected, there are legal reforms under way pertaining more to forensic psychiatry. These changes aim to strengthen the right to self- determination, including that of forensic patients. Simultaneously, more security-orientated reforms are being considered, such as broadening the use of compulsory, supervised outpatient treatment and facilitating information exchange between healthcare and other authorities, when necessary and justified by the need to prevent violent acts. Also, a comprehensive, national forensic psychiatric database is being developed and the outdated forensic unit at Kellokoski, which services Finland’s capital area, is being replaced by a new, purpose- built unit, utilizing modern standards and advances in forensic psychiatric hospital design [52, 53], thus strengthening the overall national service provision.

Ethical issues are also firmly on the forensic agenda. For instance, it is a matter of continual ethical, clinical and legal debate where the rather elusive line between care and security is drawn at any given time [39, 54, 55]. An important trend relevant to this in Finland has been—as in most Western nations-psychiatric deinstitutionalization. The number of psychiatric beds has decreased in Finland from ca. 20,000 in the 1970 to the current number of about 3500 [12]. However, as has been the case elsewhere [56–59], it has been debated in Finland whether this process should be, rather, seen as re- or transinstitutionalization through the “forensification” of psychiatric presentations previously treated within a more robust general psychiatric hospital service. What is more, the number of psychotic prisoners in Finland has increased as the number of forensic examinations, as described above, has decreased [60]. Whether there is a causal link between these phenomena, and what the reasons behind the decline in the number of forensic examinations are, is currently under investigation.

As modern challenges emerge, discussions continue between clinicians, service-users, policymakers and medico-legal authorities [35, 61–63]. Correspondingly, the search for an increasingly evidence-based forensic service system, including clinical practice and risk assessment standards, through scientific research and international collaboration [64] gives reason for optimism for forensic psychiatry in both Finland and elsewhere.

## Data Availability

Data sharing is not applicable to this article as no datasets were generated or analysed during the current study.
